# Drought impacts on children's respiratory health in the Brazilian Amazon

**DOI:** 10.1038/srep03726

**Published:** 2014-01-16

**Authors:** Lauren T. Smith, Luiz E. O. C. Aragão, Clive E. Sabel, Tomoki Nakaya

**Affiliations:** 1 University of Exeter – College of Life and Environmental Sciences Amory Building, University of Exeter, Rennes Drive, Exeter, UK, EX4 4RJ; 2 University of Bristol - School of Geographical Sciences, University Road, Clifton, Bristol; BS8 1SS; 3 Ritsumeikan University – Department of Geography 56-1 Tojiin-kita-machi, Kita-ku Kyoto, 603-8577, Japan; 4 National Institute for Space Research, Remote Sensing Division, Av. dos Astronautas, 1758, São José dos Campos, SP - 12227-010, Brazil

## Abstract

Drought conditions in Amazonia are associated with increased fire incidence, enhancing aerosol emissions with degradation in air quality. Quantifying the synergic influence of climate and human-driven environmental changes on human health is, therefore, critical for identifying climate change adaptation pathways for this vulnerable region. Here we show a significant increase (1.2%–267%) in hospitalisations for respiratory diseases in children under-five in municipalities highly exposed to drought. Aerosol was the primary driver of hospitalisations in drought affected municipalities during 2005, while human development conditions mitigated the impacts in 2010. Our results demonstrated that drought events deteriorated children's respiratory health particularly during 2005 when the drought was more geographically concentrated. This indicates that if governments act on curbing fire usage and effectively plan public health provision, as a climate change adaptation procedure, health quality would improve and public expenditure for treatment would decrease in the region during future drought events.

On average Amazonia experiences an extreme flood or drought once every ten years^1^. In a recent five year period, however, two mega droughts have struck Amazonia: in 2005 and 2010. The 2005 drought was classified as a one-in-a-hundred year event, but five years later a drought of greater magnitude struck the region again^1^. Environmental and social devastation can be caused through drought events: forest enters into water deficit causing tree mortality^2^; social impacts can include: lack of food and medical supplies, isolation of communities and health problems^3^.

During droughts, wind erosion in deforested areas causes soil particles and microbes to be blown into the air, creating and exacerbating respiratory problems, such as irritation of the respiratory tract, and triggering allergies^4^. In Amazonia, droughts can lead to over 30% increase in fire occurrence^5^,^6^. Smoke from fires tends to carry to the atmosphere fine Particulate Matter particles (PM_2.5_)^7^. These particles are extremely hazardous to human health since, when inhaled, they may reach deep in the lungs^8^, causing irritation of the throat, lungs and eyes^9^. Within Amazonia the primary location for fires is around the southern and eastern periphery: the arc of deforestation, where around 85% of fires occur^10^: emitting as much as 300–600 μg/m^3^ of PM_10_ per 24 hours^11^ and up to 400 μg/m^3^ of PM_2.5_ per 24 hours^12^ during the dry season. These particulates represent around 60% of PM released during biomass burning^7^. Measurements carried out in southern Amazonia demonstrated that exposure to PM_2.5_ have positive associations with children's respiratory health^13^. Local studies in Rio Branco, Acre State, and Alta Floresta, Mato Grosso State (Supplementary Fig. S1 for study area map) have shown a 5.6% and 2.9% increase in outpatients simultaneously with an increase of 10 μg/m^3^ PM_2.5_^14^,^15^.

Global Climate Models predict a higher probability of droughts in Amazonia by the end of the 21^st^ century in response to climate change^16^,^17^. This potential increase in drought intensity and frequency may transform Amazonia into a fire-prone system^18^ with amplified impacts upon ecosystems and humans. Despite recent demonstrations of the impact of droughts and fire on tropical ecosystems^2^,^5^,^19^,^20^,^21^,^22^, there is still a lack of large-scale and wall-to-wall assessments that preclude testing how these droughts would affect tropical population's health.

The availability of operational satellite-derived rainfall from the Tropical Rainfall Measuring Mission satellite (TRMM)^23^; active fires and aerosol from Terra/MODIS satellite^24^,^25^; and deforestation rates^26^ from the National Institute for Space Research (INPE) datasets; together with the geo-spatial information about socio-economic indices from the Brazilian Institute of Geography and Statistics (IBGE) and hospitalisations from the Brazilian Health System (SUS), provide a unique opportunity to quantify the sensitivity of children's respiratory health to environmental changes induced by recent droughts in the whole Brazilian Amazon. The relationships between respiratory health and recent Amazonian drought events may provide an approximation of the expected responses of health to future climate conditions. Therefore, in this study we tested whether the incidence of respiratory diseases during two major droughts (2005 and 2010) was statistically dependent on drought-related environmental changes and socio-economic factors, by using Geographically Weighted Poisson Regression models (GWPR) for the whole Brazilian Amazon.

## Results

**Figure 1:**
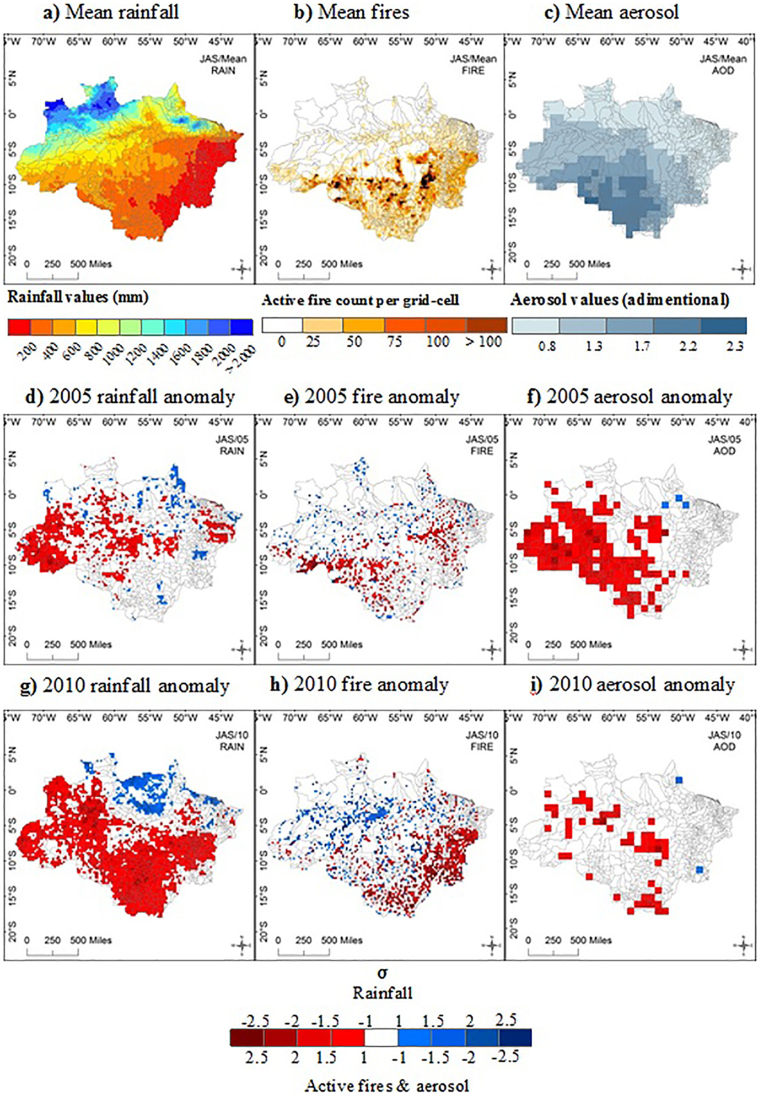
Standardised JAS anomalies as a departure from the 2001–2010 mean. (a) mean rainfall values (mm) (2001–2010), (b) mean active fire count (2001–2010), (c) mean aerosol levels (2001–2010), (d) standardised rainfall anomalies for 2005, (e) standardised active fire anomalies for 2005, (f) standardised aerosol anomalies for 2005, (g) standardised rainfall anomalies 2010, (h) standardised active fire anomalies 2010, (i) standardised aerosol anomalies for 2010. The anomalies are normalised by the standard deviation of the time-series (2001–2010) for each grid-cell. Anomalies were generated using ENVI 4.8 and ArcGIS 10.

To establish the extent and duration of the drought conditions we calculated rainfall, active fire, and aerosol anomalies as a departure from the 2001–2010 long-term mean (Methods & Supplementary Fig. S2). In 2005 large negative rainfall anomalies (≤−1 σ) affected the south-west part of the Amazon. These anomalies peaked in the dry season months of July, August and September (JAS) when 41% of municipalities had at least one anomalous grid-cell (Figure 1). Like 2005, the intensity of the negative rainfall anomalies in 2010 increased in JAS (anomalies ≤ −1 σ) (52.3% of all grid-cells) (Figure 1).

During 2005 a clear anomalous increase in active fire frequency was observed in the epicentre of the drought during JAS (Figure 1). In JAS 2010 the intensity of active fires around the arc of deforestation increased following the spatial pattern of drought affected areas. Areas in southern Maranhão, Tocantins and Mato Grosso States were subject to the greatest positively anomalous grid-cells (≥1 σ) (72.8%) (Figure 1). Overall, the cumulative number of active fires was 39% and 36% higher than the 2001–2010 mean during 2005 and 2010 droughts, respectively.

During the 2005 and 2010 droughts, the JAS period saw 246 and 458 of 807 municipalities being classified as drought affected in this study, (Supplementary Methods & Fig S3). For 31.3% (77) of the municipalities affected by the 2005 drought, the total number of hospitalisations increased between 1.3% and 180.8%, in comparison to the ten-year mean. Capixaba in Acre State was the municipality with the largest increase in hospitalizations during the 2005 drought (180.8%). Similarly, for 43.0% (197) of the municipalities affected by the 2010 drought, the total number of hospitalisations increased between 1.2% and 267%, in comparison to the ten-year mean. For the 2010 drought, the highest increase in total hospitalisations in relation to the ten year mean was in Tocantins State, with the municipality of Fátima displaying a 267% increase in hospitalisations. Our results confirm the association between increases in emergency room visits and hospitalisations for respiratory diseases and periods of high fire counts previously observed at the local scale^27^,^28^.

**Figure 2:**
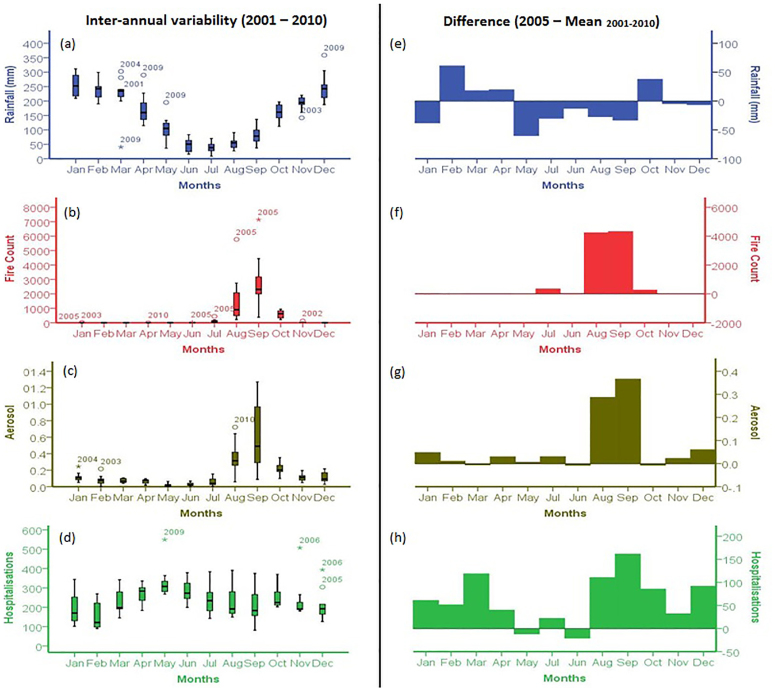
Temporal trends in Acre 2005. The right column shows average monthly inter-annual variability (2001–2010) for Acre State, while the left column represents the difference between 2005 values for Acre State compared to the mean values. (a) average rainfall, (b) cumulative active fires, (c) average aerosol values, (d) number of hospitalisations, (e) difference in average rainfall, (f) difference in cumulative active fires, (g) difference in average aerosol, (h) difference in the number of hospitalisations.

This analysis also brings to light the fragility of population health to drought-associated impacts at the epicentre of the 2005 drought. This statement becomes clear when analysing the seasonality of health data in Acre State alone, during the 2005 drought. The total number of hospitalisations for respiratory diseases increased 88% compared to the same period in 2004. Moreover, this value was 54% larger than the ten year mean. Throughout 2005, JAS also accounted for the greatest number of hospitalisations in Acre despite a general trend of hospitalisations peaking at the end of the wet season (Figure 2).

**Figure 3:**
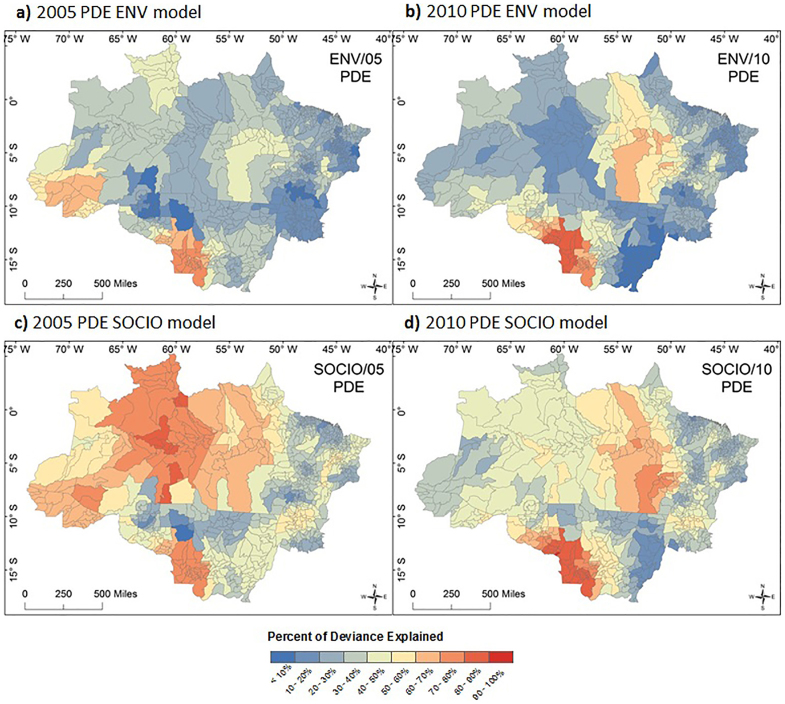
Percent of deviance explained. (a) goodness-of-fit for the ENV model 2005, (b) goodness-of-fit for the ENV model 2010 and 2010, (c) goodness-of-fit for the SOCIO model 2005, (d) goodness-of-fit for the SOCIO model 2010. Red shades show an increase in the goodness-of-fit of the model. Percent of deviance explained was generated in GWR4 and mapped using ArcGIS 10.

To examine the spatial relationship between respiratory diseases in children aged under-five and drought conditions, we use a local model, named Geographically Weighted Poisson Regression (Methods, Supplementary Methods and Table S1 for further information). As a baseline for comparing the model for drought years, we first run the model using the ten-year average values (2001–2010) for each variable associated with drought events (MEAN model). (Supplementary Fig. S5–6). The MEAN model for environment variables only (ENV) showed a goodness-of-fit, a similar measure of coefficient of determination (% of deviance explained, PDE), of 32%, increasing to 67% when including Human Development Index (HDI) and population density as independent socio-economic variables (SOCIO). In the MEAN ENV model deforestation exhibits significantly positive z values in most municipalities in the Brazilian Legal Amazon compared with the other variables. By including socio-economic variables to the analysis (MEAN SOCIO) HDI becomes the dominant variable affecting hospitalisations for respiratory diseases: suggesting development levels are more significant in the development of respiratory health problems than environmental variables in non-drought years. An initial analysis using an ENV model for drought years showed an increase in goodness-of-fit of 56% for 2005 in south-western Mato Grosso, Acre, and Amazonas States and 52% for 2010 in south-western Mato Grosso and eastern Rondônia States (Figure 3 and Supplementary Fig. S7). Aerosol exhibited the strongest significant positive relationship. The other z values of local coefficients are significant but with much lower magnitude than aerosol. This indicates that aerosol may be the primary force driving increases in respiratory diseases during droughts (Supplementary Figs S8–9). In a subsequent analysis, we added HDI and population density as independent socio-economic variables in our geographical model. When including these factors (SOCIO), the goodness-of-fit increased in the drought affected municipalities. We observed that 68% of deviance was explained by the SOCIO model in western and central Amazonia for 2005. For 2010, however, the amount of deviance explained (60%) by the SOCIO model remained spatially similar to the ENV model (Figure 3).

**Figure 4:**
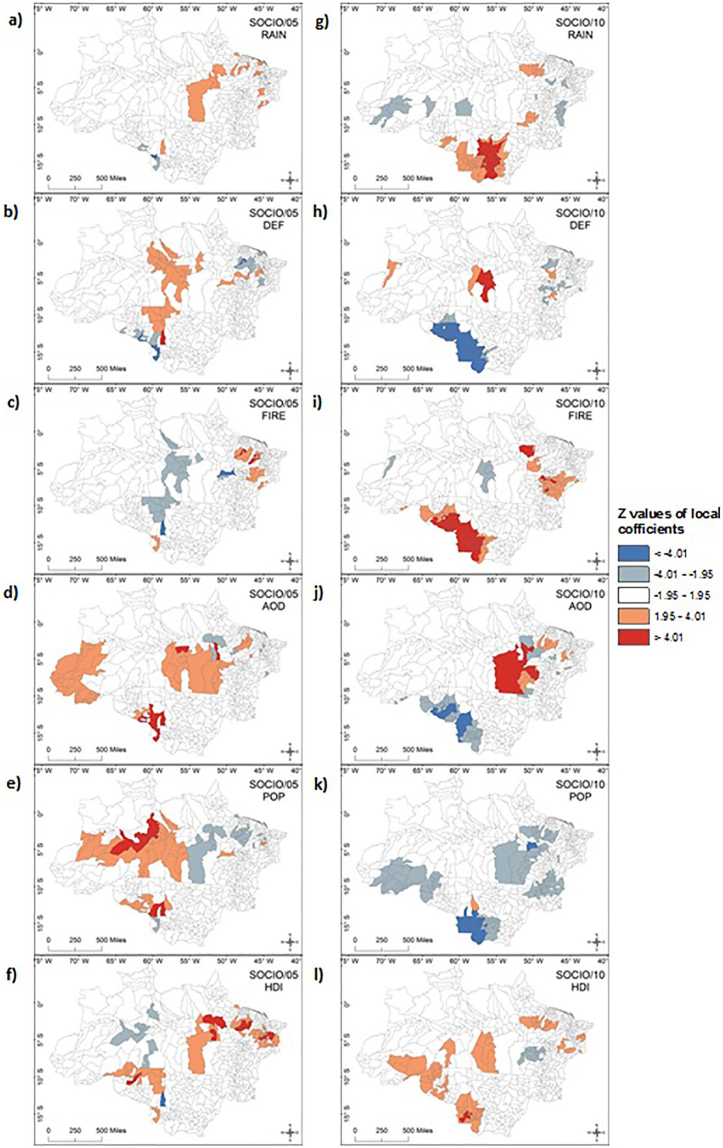
Significant values with a confidence level greater than 95% for the SOCIO model in drought affected municipalities. Non-significant municipalities and municipalities that were not affected by the droughts are masked out. (a) 2005 rainfall, (b) 2005 deforestation, (c) 2005 fire, (d) 2005 aerosol, (e) 2005 population density, (f) 2005 HDI, (g) 2010 rainfall, (h) 2010 deforestation, (i) 2010 fire, (j) 2010 aerosol, (k) 2010 population density, (l) 2010 HDI. Red shades show positive z values of local coefficients, while blue shades show negative estimates of local coefficients - the darker the shade the stronger the relationship. Z values of local coefficients were generated in GWR4 and mapped using ArcGIS 10.

The z values of local coefficients generated in the GWPR analysis indicated that respiratory diseases in 2005 and 2010 were primarily influenced by aerosol and HDI. Aerosol showed a significantly positive association with respiratory diseases in 28% of the drought effected municipalities with estimates of local coefficients as high as 13.2 in Maranhão, while significantly negative associations were observed in 4% of drought effected municipalities. In 2010 significantly positive associations were found in 6% of drought affected municipalities: 17% were statistically negative. 38% of drought affected municipalities in 2005 showed statistically positive associations with HDI and 3.6% showed statistically negative associations. In 2010, the percentage of affected municipalities was 19% (positive) and 4% (negative) (Figure 4; Supplementary Fig. S10). The z values of local coefficients also suggested that aerosol affected a larger proportion of the Brazilian Amazon (40 more municipalities with statistically positive associations) in 2005 in comparison to 2010 despite the latter being an event of larger extent. Examining only areas that were severely affected by rainfall shortage (anomalous rainfall pixels ≤ −2 σ), aerosol was once again the key variable parameter around Acre State (Supplementary Fig. S11). Aerosol was significant in both ENV and SOCIO analyses, highlighting the importance of its influence on respiratory diseases in drought years compared with non-drought years where it was non-significant or showing predominantly significantly negative association as shown in the MEAN models.

## Discussion

Our data demonstrated that peak hospitalisations in Acre generally occur at the end of the wet season, however during the 2005 drought, this peak was displaced and in accordance with the fire season: indicating a direct effect. Previous analysis for Mato Grosso State also showed higher monthly hospitalisation rates at the end of the wet season and the interim period between seasons^29^. This is because of higher levels of humidity during the wet season, leading to an increase in funguses and mites that are powerful allergens for the occurrence of respiratory diseases^30^. Alternatively this increase could be related to the start of the school term, when children enter in contact with others, increasing the risk of exposure to infection, or aligned with operational factors such as hiring physicians during this time: allowing more patients to be treated.

High concentrations of smoke were recorded in Rio Branco, during September 2005^31^, which could explain the local parameter estimate for aerosol in this region. HDI was significantly positive in drought affected municipalities in Rondônia, western Mato Grosso, Pará, and Maranhão States, but not around the epicentre of the 2005 drought in Acre. Significantly negative areas of HDI are observed in Amazonas. The associations between respiratory diseases and socio-economic status are complex, thus a more detailed investigation into socio-economic status and respiratory diseases is needed to provide possible explanations. However, it could be attributed to the Hygiene Hypothesis that suggests the population in more developed areas may be more susceptible to disease because they are less exposed^32^. Population density was significant in some drought affected municipalities but none around the epicentre of the drought. Moreover, this variable was not significant at the epicentre of the 2005 drought in Acre. Rainfall, active fires and deforestation were not significant in the majority of drought affected municipalities.

Despite the 2010 drought being of greater magnitude than the 2005 drought, the impact on respiratory diseases was not as severe. Similarly to the 2005 drought event, HDI had the greatest influence, with larger areas experiencing significantly positive z values of local coefficients. The large coverage of significantly positive HDI in both years could be due to the Hygiene Hypothesis. The strength of HDI in 2010 in relation to the environmental variables may be due to the large spatial extent of the drought which disguised localised environmental impacts, similarly to the MEAN SOCIO, compared to 2005 when the drought was concentrated around Acre State. Aerosol was significantly and positively related to respiratory disease incidence in southern Pará. Elsewhere, significantly negative z values of local coefficients were observed, particularly along the eastern and southern edge of the region, and pockets in Tocantins: highlighting locations where aerosol loads do not increase the number of hospitalisations. This may be because the population living in these locations are accustomed to air pollution exposure as this area sees most fires occurring year on year. Moreover, the 2010 drought experienced fewer fires and anomalous aerosol loads compared to 2005 (Figure 1). The other local parameter estimates were close to zero suggesting they did not affect respiratory health as greatly (Figure 4).

This is the first analysis of the impacts of drought on respiratory health of children under-five years at the scale of the whole Brazilian Amazon. Despite the wide trend of respiratory diseases peaking at the end of the wet season, drought condition exacerbates the incidence of respiratory diseases in children during the dry season. We suggest that the increase in respiratory diseases during the 2005 drought was driven mainly by aerosol and HDI. Conversely, HDI overcame the impact of aerosol during the drought of 2010: due probably to the decrease in aerosol emissions associated to a reduction of 1.9% in fire incidence in 2010 in relation to 2005. This study brings a new dimension into the debate around climate and environmental change impacts in tropical nations. Feedbacks between climate change and land use conversion are likely to reduce rainfall in Amazonia^33^, with important implications for agriculture and water supply^34^. We can now conclude that in the Brazilian Legal Amazon not only forests^2^ are threatened by drought^2^ and fires^35^, but also human populations exposed to health-hazardous agents. It is encouraging, however, that by efficiently enforcing fire control legislation, policy makers could, with a single action, mitigate fire impacts on ecosystems^35^ and on human health. However, adaptation measurements must be pursued in terms of establishing hospitals in critical areas and planning for greater demand on health services during drought periods. These policies, together, would ensure better life quality for local populations and potentially minimize monetary and life costs under a scenario of increased future drought frequency.

## Methods

### Environmental anomalies

Environmental anomalies have been calculated based on the reference mean of 2001–2010 for consistency with the period MODIS data is available.

To quantify the spatial and temporal extent of the drought, rainfall surfaces of Tropical Rainfall Measuring Mission (TRMM) (3B43-v6) data at spatial resolution of 0.25° were grouped at 3-monthly intervals to show seasonal differences. Aragão, et al^5^. validated the TRMM dataset evidencing the relationship between satellite-derived rainfall and rain gauges data in Amazonia. Anomalies for 2005 and 2010 were calculated based on the departure from the 2001–2010 mean (TRMM 2001–2010) and normalised by the standard deviation (σ 2001–2010). This was done for each year (*y*), each quarter (*q*) and at a cell by cell (*i, j*) level^5^ (equation 1). 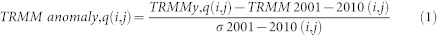
Anomalies for active fires were calculated using hot pixel density based on the accumulation of active fire pixel counts for each quarter. Hot pixels are indicators of fires, which although may be underestimated, do allow for patterns over time to be seen^5^. We used monthly active fire data for the period between 2001–2010 produced by the University of Maryland (UMA) from the Brazilian Institute for Space Research, Queimadas project database. These anomalies were calculated following a similar method to the TRMM data (Equation 1).

Aerosol Optical Depth/Thickness data, (AOD/AOT, Optical Depth Land and Ocean at 0.55 microns, MOD08_M3 collection 051), referred to in the text as aerosol, was used for the same ten year period. Values vary between −1 and 5 (adimentional), the higher the value the more concentration of particles in the air. Maximum values for Aerosol surfaces were grouped at 3-monthly intervals to correspond with rainfall anomalies. These were used to calculate anomalies using a similar method to Equation 1. In addition, environmental data were aggregated to municipalities to allow for comparison with the number of hospitalisations and use in Geographically Weighted Poisson Regression (GWPR).

### Datasets

Deforestation data was obtained from INPE PRODES project. Unlike the other environmental variables measurements for deforestation is an annual count from August to August.

Databases of the Sistema de Informações Hospitalares, SIH/SUS (Hospital Information System) of the Brazilian Ministry of Health were utilised to obtain information regarding hospitalisation data for respiratory diseases. Chapter X, Diseases of the Respiratory System, of the International Classification of Diseases revision 10 was used (coded from J00 to J99), for people living in the Brazilian Amazon between 2001–2010. Hospitalisation data for all children aged under-five years consisted of: Hospital admissions (paid authorisation for hospitalisations), municipality of residence, year of admission, and month of admission. Cases were selected based on residence rather than hospital attended; this ensures a better representation of the spatial distribution of the exposed population to the environmental factors.

It is important to also include social indicators as they also provide risk factors for respiratory disease. Fijan system in Brazil, have produced local HDI values per municipality based on income and employment, education and health variables.

Population data for each municipality in the Amazon was obtained from the Brazilian Institute for Geography and Statistics (Instituto Brasileiro de Geografia e Estatística, IBGE).

### Spatial analysis

The local model, GWPR, has been used to identify relationships between drought events and respiratory diseases because the environment varies throughout the Amazon and health is known to vary spatially. The details of the model is described elsewhere^36^. The model of GWPR is shown in equation 2. 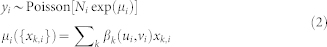
where *y_i_*, *μ_i_*, *x_k,i_* and *N_i_* are, respectively, dependent variable (the total number of children aged under-five who were hospitalised for respiratory diseases), the linear predictor, *k*th independent variable including the constant term and the offset variable corresponding to population size at risk (defined as population aged under-five) at the location *i*. It should be noted that the estimated risk of child hospitalisation for respiratory disease at the location *i* is given by the term of exp(*μ_i_*). (*u_i_*,*v_i_*) is the x-y coordinate of the *i*th location; and coefficients *β_k_*(*u_i_*,*v_i_*) are assumed to be smoothly varying conditional on the location. In the case of SOCIO model, the linear predictor can be rewritten with the names of independent variables as (equation 3): 
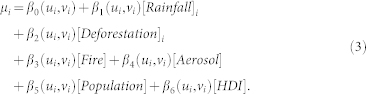
The estimates of the local coefficients at the location *i* are obtained by fitting a usual Poisson regression model to the data subset around the regression point *i* with a geographical weighting function. The standard errors of estimated local coefficients can be derived by local regression theory. A descriptive measure of goodness-of-fit for Poisson regression is percent of deviance explained: 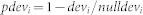
where *dev_i_* is the deviance of the fitted model and *nulldev_i_* is the deviance of the null model having only a constant term. The equivalent measure for the local fitting at each location that can be derived by the local weighting of the deviance of fitted and null models. The estimated values of local coefficients and the local goodness-of-fit measures can be mapped to assess the spatial variability of relationships between the dependent and independent variables as seen in Figures 3 and 4.

To some degree, overdispersion tends to be present in the vast majority of count data; this is the case with hospitalisation count data for children under-five in the Legal Amazon. To manage this, standard errors have been adjusted based on the quasi-likelihood theory to produce adjusted z values (equation 4). 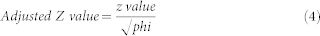
where, *phi* is the deviance divided by the degrees of freedom.

To identify significant locations the Bonferroni correction method has been used which identified z-values of ±4.01 and ±1.95 based on the number of regression points 807 with threshold p-value of 0.05 (Figure 4, and Supplementary Figs. 5–11). Z values of location specific coefficients (equation 5): 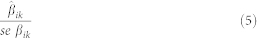
are mapped in Figure 4 and Supplementary Figures 5–11. The derivation of the standard error (se) of GWPR is described elsewhere^36^, and the terms of equation 5 are described in the text detailing equation 2.

## Author Contributions

L.E.O.C.A., L.T.S. and C.E.S. designed the study. L.T.S. carried out analysis with the support of T.N. for the GWPR methodology. C.E.S., L.E.O.C.A. and T.N. interpreted the results and revised the paper. L.T.S., L.E.O.C.A. and C.E.S. wrote the paper.

## Supplementary Material

Supplementary Information Supplementary Information
