# Supplementary material for: Drought impacts on children's respiratory health in the Brazilian Amazon
Source: Sci Rep. 2014 Jan 16;4:3726. doi: 10.1038/srep03726 (PMC3893650; doi:10.1038/srep03726)
Supplement: Supplementary Information [file srep03726-s1.pdf]

Drought impacts on children’s respiratory health in the Brazilian Amazon

Lauren T. Smith, Luiz E. O. C. Aragão, Clive E. Sabel, Tomoki Nakaya.

Supplementary Methods

Addition information detailing the criteria used to define drought affected municipalities, the GWPR model selection, and the choice of datasets.

Supplementary Table 1

GWPR model summary, AICc values and percent of deviance explained.

Supplementary Figures

Additional information detailing a location map, rainfall and fire anomalies, drought affected areas, areas of deforestation, percent of deviance explained in drought affected municipalities, z values of local coefficients for the ENV model for the study area and for drought affected areas, and z values of local coefficients for the SOCIO model for the study area and for severely affected municipalities.

Supplementary Notes

Additional references from supplementary methods.

Supplementary Methods

Defining Amazon Drought: Drought conditions in Amazonia are referred to as months with 100 mm or less of rainfall because the forest enters into water deficit[1,2]. Critical areas of drought conditions within the basin were identified as municipalities with at least one anomalous pixel and ≤ 300 mm of cumulative rainfall per quarter to ensure drought conditions were experienced and it was not the normal climate for the municipality (Supplementary Fig. S3). This definition for drought was used for exploratory analysis, rather than included in the model.

Geographically Weighted Poisson Regression (GWPR): We had a prior understanding of the droughts in the Legal Amazon and so a spatially explicit model for 2005 and 2010 was carried out to understand the consequences of the drought events. Previous analysis of the mean values of each variable for the 2001 to 2010 period helps to show the impacts on children's respiratory health during the droughts (Supplementary Fig. S5-6). The research question was to assess the impacts of drought conditions on respiratory diseases in children aged under-five. To address this, data has been analysed using the GWR4 software[3] with an adaptive kernel weighting, and golden section search for the optimal bandwidth size by minimising Akaike information criterion (AICc). The adaptive kernel was selected to account for the variation in municipality size. Better models have lower AICc values, with the general rule of a difference of at least three[4], Table S1 shows the local model (GWPR) is performing better than the global model. A global model summaries data for a whole region, assuming stationary processes, and as such are location independent. Whereas a local model takes into account variations that occur within a region and thus produces different values in different locations within the region. The golden search procedure gave the optimal bandwidths of 60 for JAS05 social, and 77 for JAS10.

Geographically Weighted Regression was used for three main reasons. It is based on the traditional regression framework which most people are familiar with so it is fairly easy to understand the basics of the model[4]. Also, it incorporates local level data rather than 'global' data which assume to represent the situation in every part of the study area. If the study region had little variation a global model would be acceptable, however the Legal Amazon is a vast area with varying land use, climate, population structure, and development. Moreover, incorporating the Poisson regression into the analysis (GWPR) it provides a more appropriate basis for analysing areal data where observed counts may include low count numbers, which can arise when working with health data [5].

An important advantage of using GWPR other than those discussed above, is that it allows a combination of geographically varying and geographically constant parameters in the model [6]. Although this function was not utilised in this analysis it provides the opportunity for future work to be carried out using the same method if both geographically varying and constant variables are needed to be included. Limitations of the method do exist, however these are related to units of observation. It can sometimes be difficult to predefine categories, and result in maps having little meaning [6], for overcoming this problem, z-values are mapped so comparisons can be made.

Use of datasets: Count data of hospitalisations for all children aged under 5 years was used for the number of hospitalisations for respiratory diseases per month and per municipality[7]. Monthly rainfall (mm)[8] (RAIN) data has been included because it provides information on where the droughts have occurred using the criteria discussed in Method S1. Deforestation rates (km^2)[9] (DEF) are used to identify possible locations of where fires may occur as well as locations where increased aerosol loads may be due to exposure soil and the particles released during the process. Active fire counts[10] (FIRE) provide us with the opportunity to examine the relationship between drought events and fire occurrence as well as suggesting locations within the Legal Amazon where air pollution may be higher thus locations with greater risk to respiratory diseases. The final environmental variable, aerosol optical depth[11] (AOD) was used to assess actual levels of air pollution across the Legal Amazon as studies elsewhere (discussed in the main text) have found associations between air pollution and respiratory diseases. Including social factors, population density[12] (POP) was used as studies have suggested variability in asthma prevalence could be due to population density[13]. Also it can be used as an urbanisation index which may account for some kinds of urban air pollution. HDI[14] (HDI) was used as an indicator for socio-economic levels and development of each municipality as studies in Latin America on asthma have identified low socio-economic level being associated with the disease[15,16].

Tables

Table S1| Summary of model results.

	Model	AICc	Percent of Deviance explained
2005	Global Environmental	14975.33	0.01
	Local Environmental	6899.01	0.56
	Global Social	14248.12	0.056
	Local Social	5300.90	0.68
2010	Global Environmental	12554.14	0.08
	Local Environmental	6725.82	0.52
	Global Social	12214.44	0.11
	Local Social	5727.31	0.60

Figures

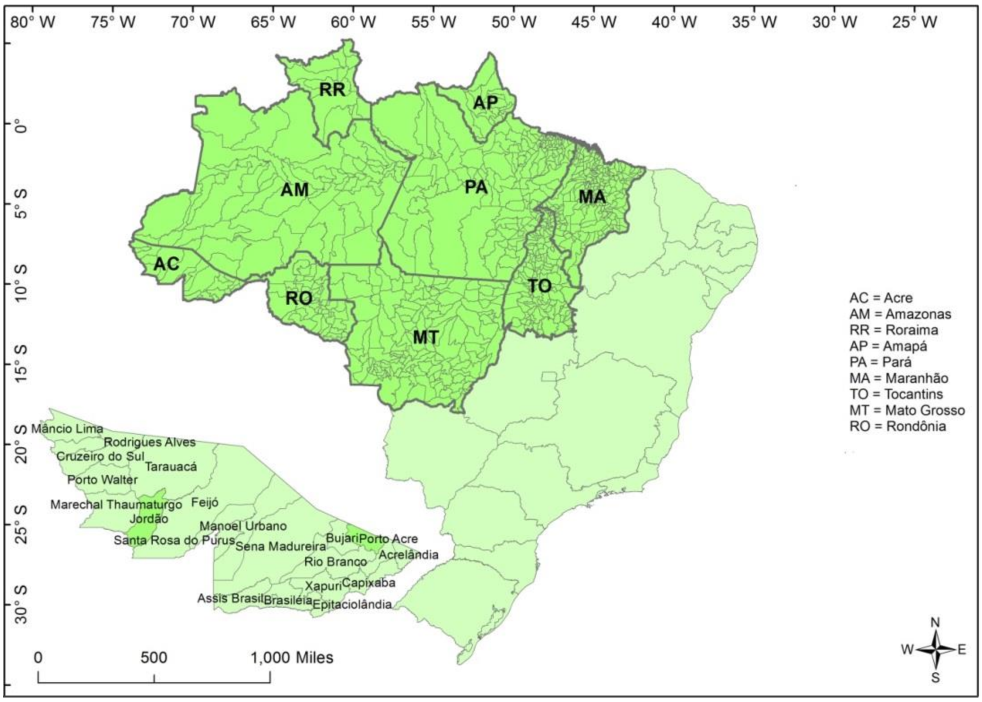

Figure S1| Location Map. Brazilian Legal Amazon boundary in darker shade of green and municipalities discussed within the text. The location map was generated in ArcGIS 10.

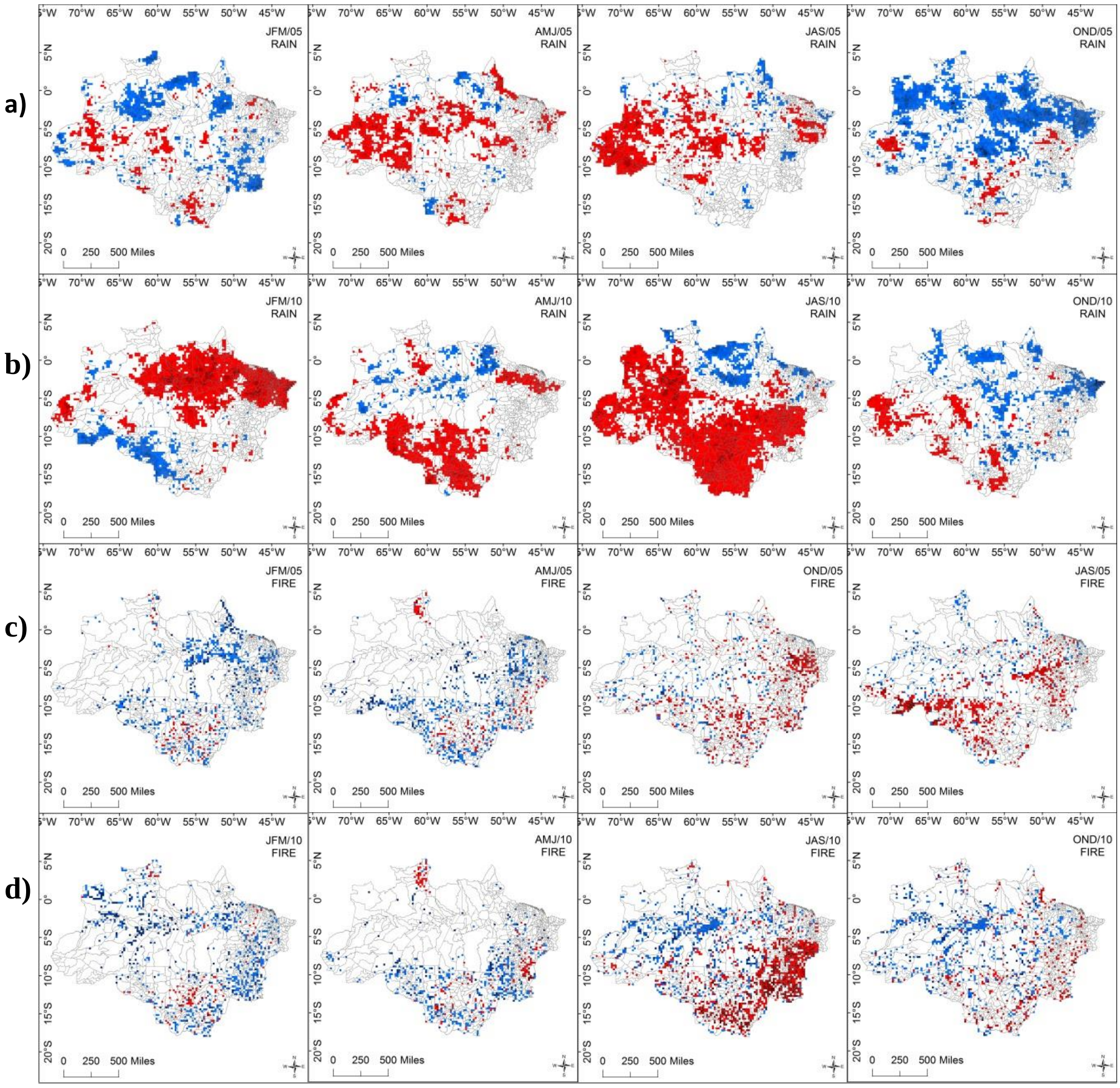

Figure S2 | Standardised seasonal anomalies as a departure from the 2001-2010 mean. a, rainfall anomalies for 2005, **b** rainfall anomalies for 2010. In both **a** and **b** red indicates areas of negative rainfall anomalies and blue areas indicating areas of positive rainfall anomalies. **c**, active fire anomalies for 2005, **d,** active fire anomalies for 2010. Positive active fires anomalies for both **c** and **d** are indicated by red areas and blue areas indicating areas of negative active fire anomalies. Anomalies were generated using ENVI 4.8 and ArcGIS 10.

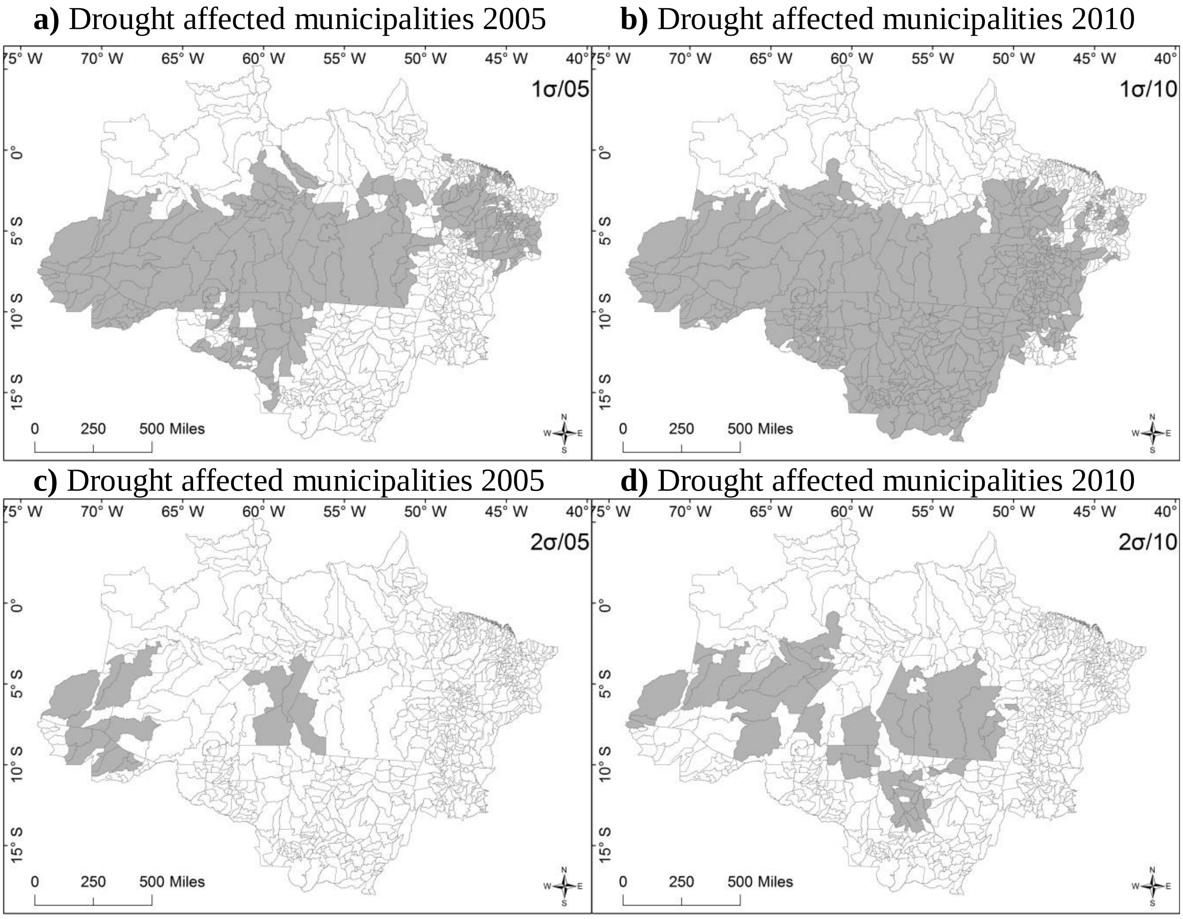

Figure S3| Drought affected areas. a, 2005 **b,** 2010 **c**, Severely hit municipalities in 2005 **d**, severely hit municipalities for 2010. Grey shaded areas illustrate the drought affected municipalities. These maps were produced using ArcGIS 10.

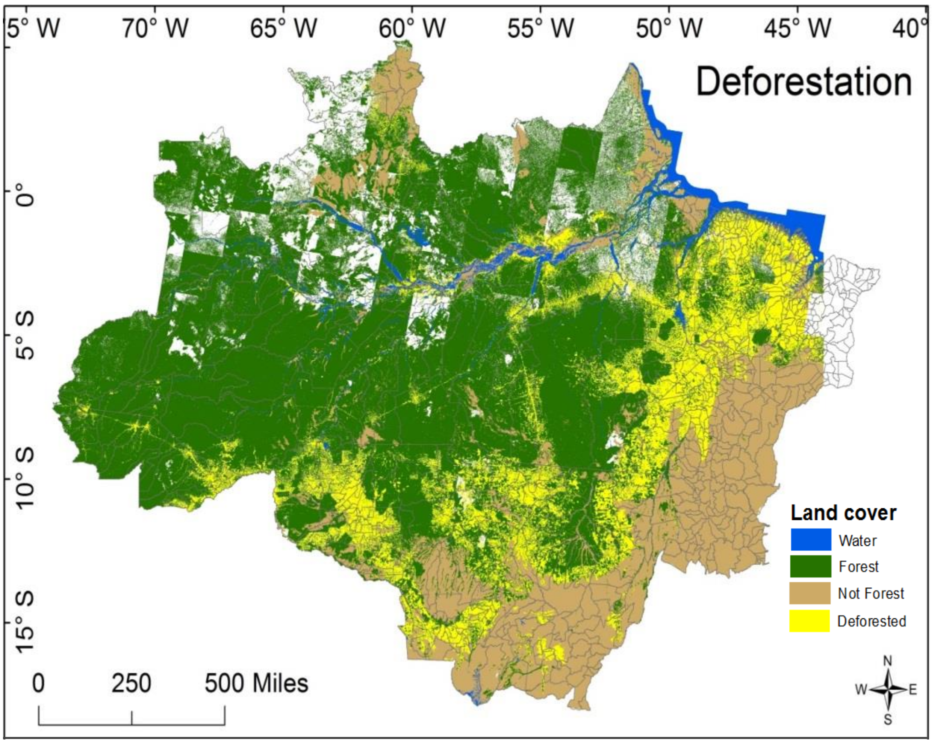

Figure S4| Location of deforestation. Land cover was produced using ArcGIS 10.

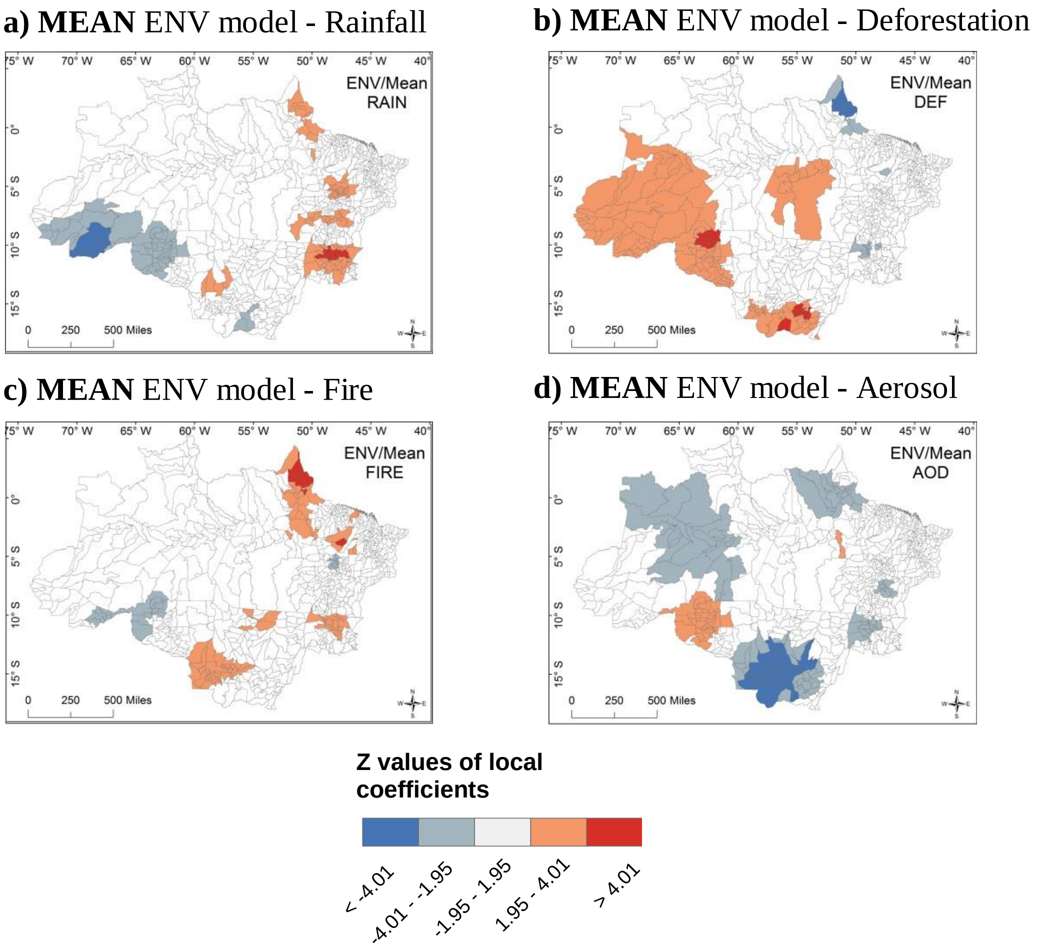

Figure S5| Significant values with a confidence level greater than 95% for the MEAN ENV model for the whole Brazilian Legal Amazon. Non-significant are masked out. **a** rainfall, **b** deforestation, **c** fire, **d** aerosol. Red shades show positive z values of local coefficients, while blue shades show negative z values of local coefficients - the darker the shade the stronger the relationship. Z values of local coefficients were generated in GWR4 and mapped using ArcGIS 10.

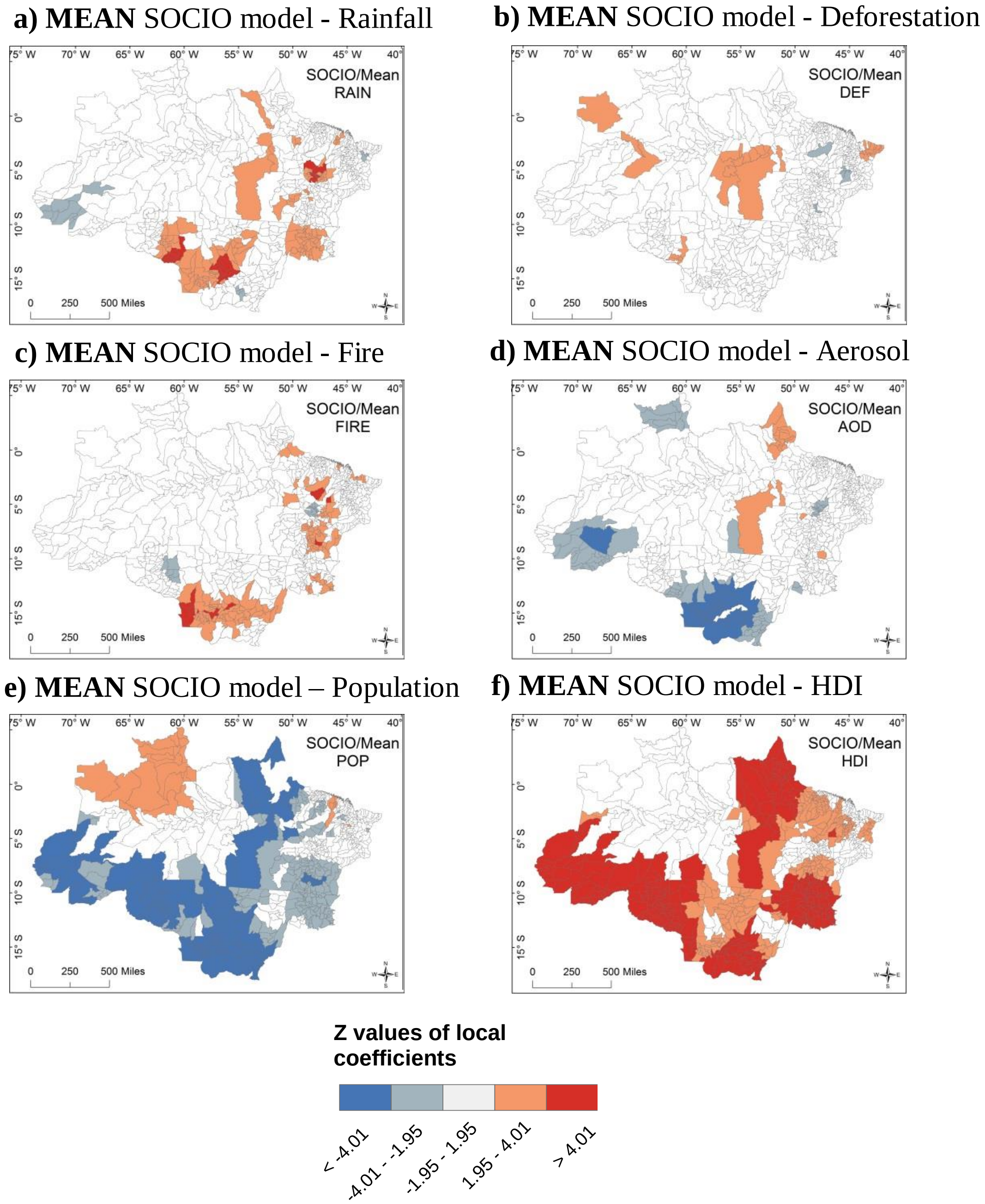

Figure S6| Significant values with a confidence level greater than 95% for the MEAN SOCIO model for the whole Brazilian Legal Amazon. Non-significant are masked out. **a** rainfall, **b** deforestation, **c** fire, **d** aerosol, **e** population, **f** HDI. Red shades show positive z values of local coefficients, while blue shades show negative z values of local coefficients - the darker the shade the stronger the relationship. Z values of local coefficients were generated in GWR4 and mapped using ArcGIS 10.

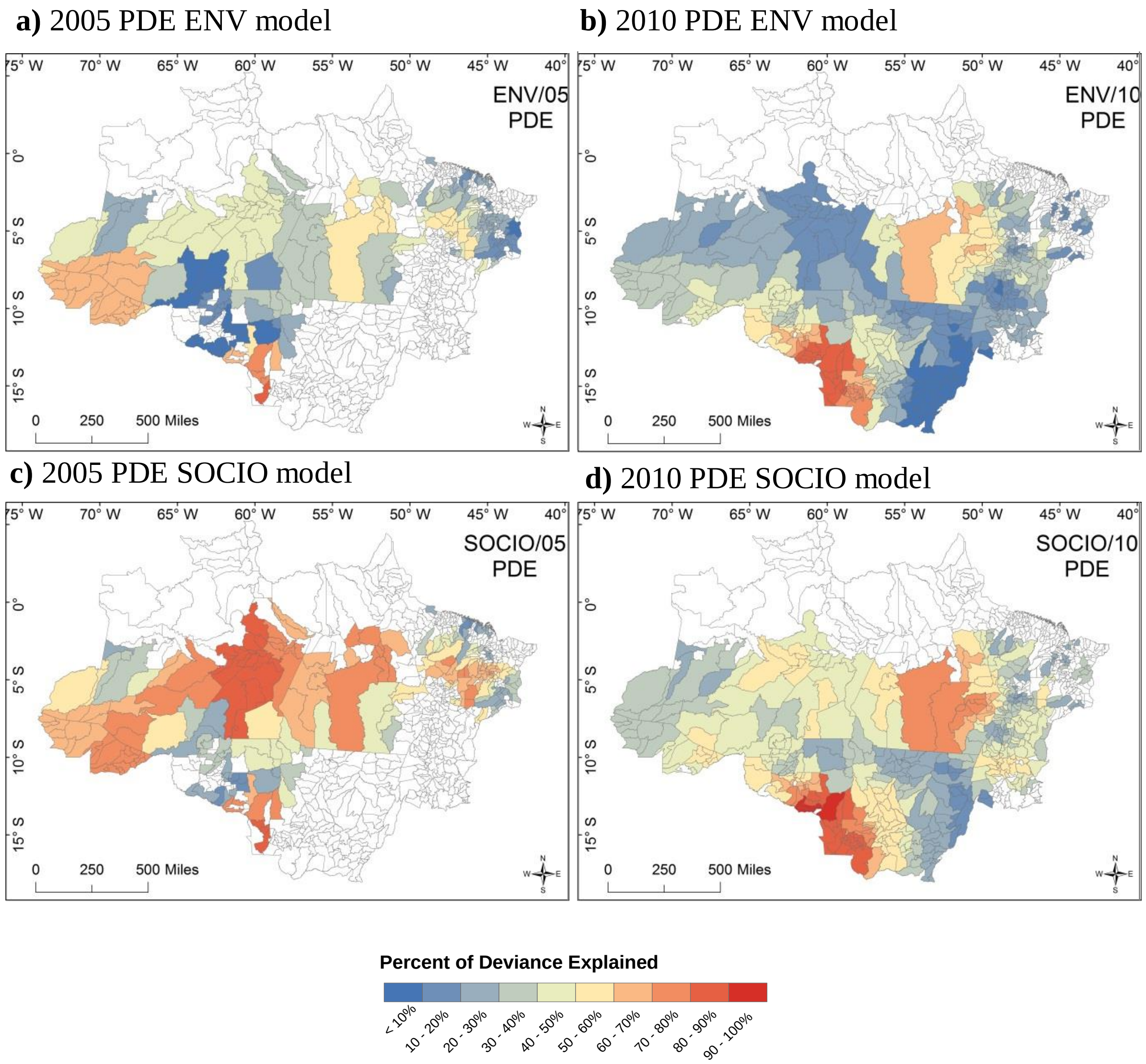

Figure S7| Percent of deviance explained in drought affected municipalities. a goodness-of-fit for the ENV model 2005, **b** goodness-of-fit for the ENV model 2010, **c** goodness-of-fit for the SOCIO model 2005, **d** goodness-of-fit for the SOCIO model 2010. Red shades show an increase in the goodness-of-fit of the model. Percent of deviance was generated in GWR4 and mapped using ArcGIS 10.

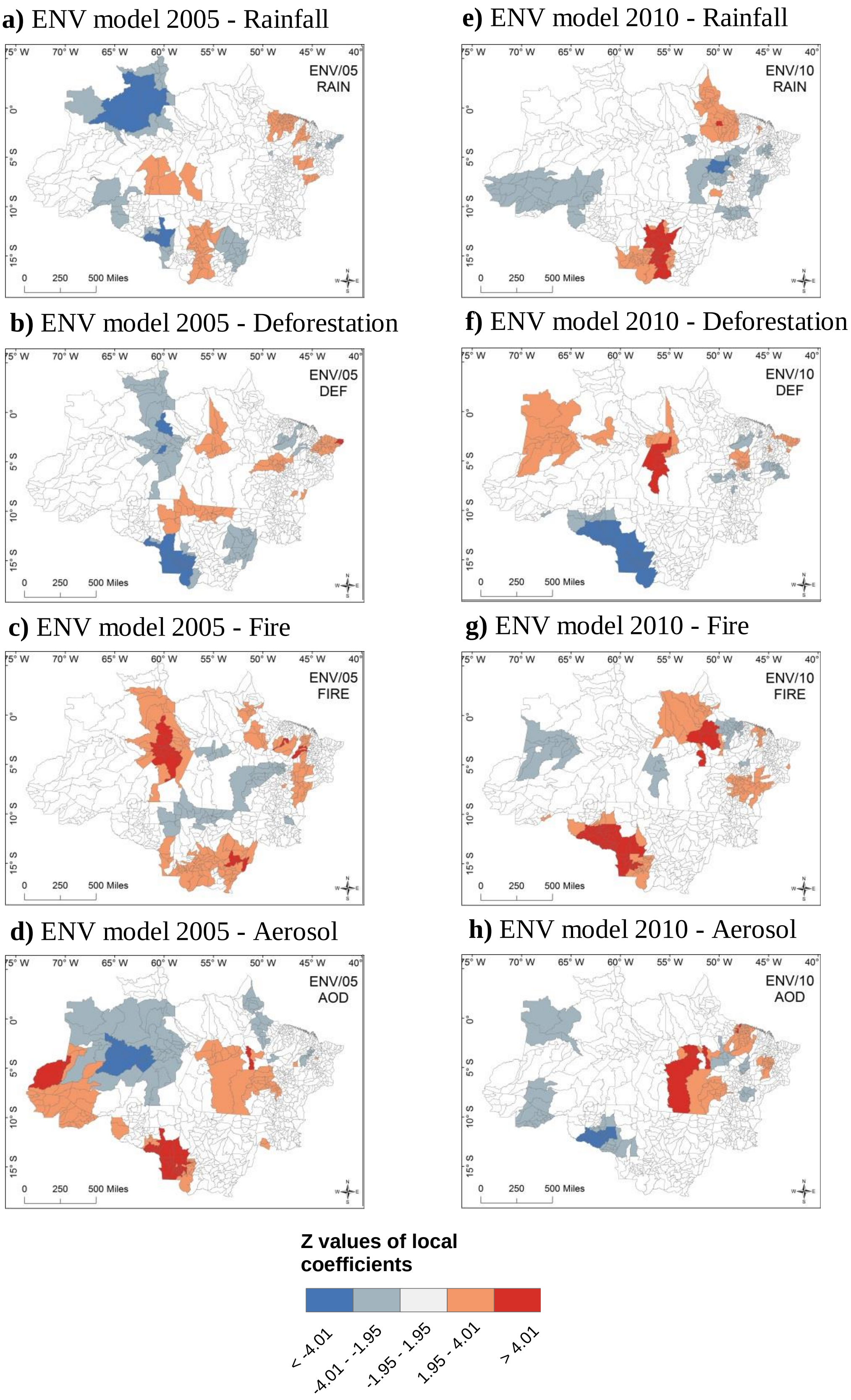

Figure S8| Significant values with a confidence level greater than 95% for the ENV model for the whole Brazilian Legal Amazon. Non-significant are masked out. **a** 2005 rainfall, **b** 2005 deforestation, **c** 2005 fire, **d** 2005 aerosol, **e** 2010 rainfall, **f** 2010

deforestation, **g** 2010 fire, **h** 2010 aerosol. Red shades show positive z values of local coefficients, while blue shades show negative z values of local coefficients - the darker the shade the stronger the relationship. Z values of local coefficients were generated in GWR4 and mapped using ArcGIS 10.

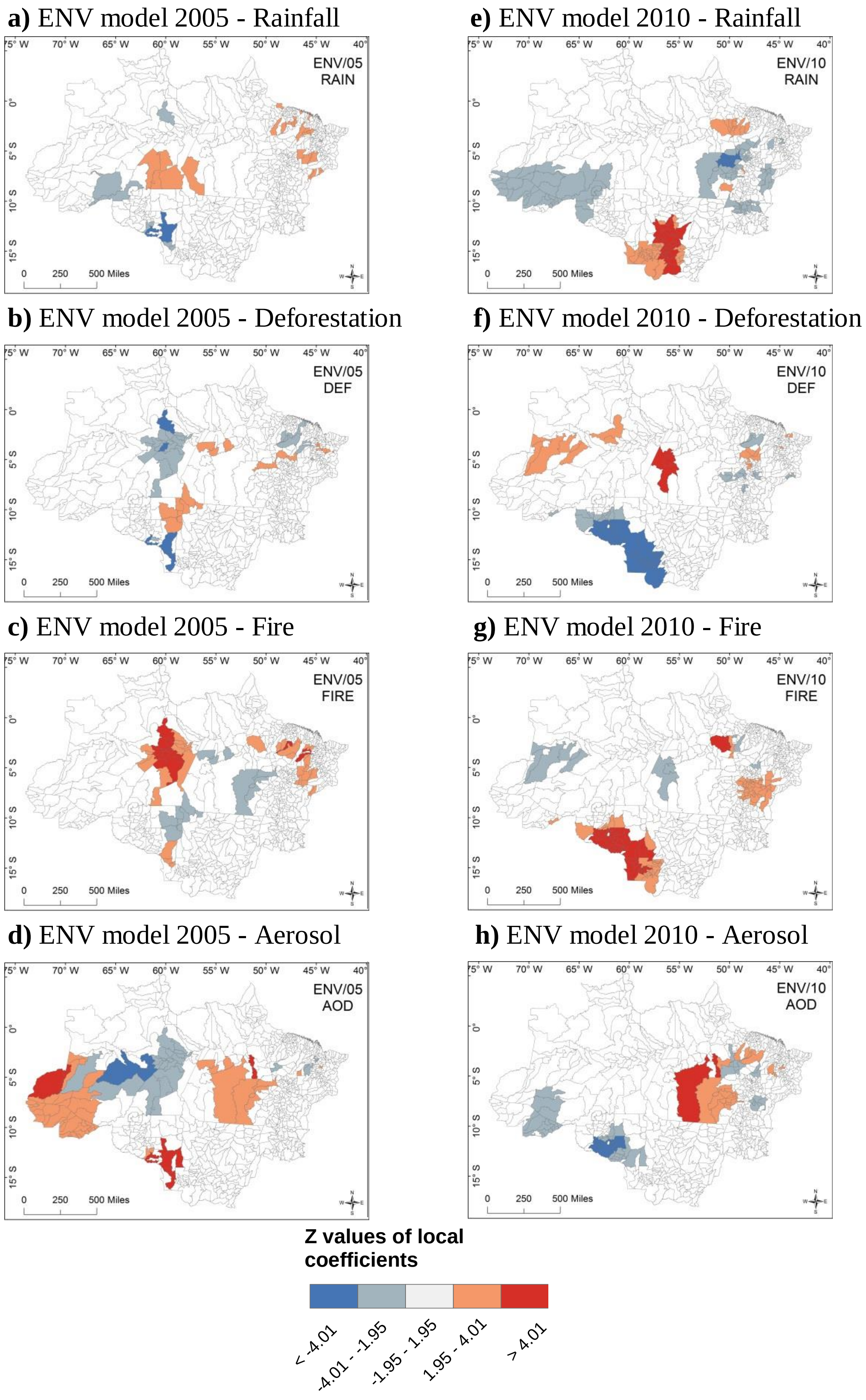

Figure S9| Significant values with a confidence level greater than 95% for the ENV model n drought affected municipalities. Non-significant municipalities and municipalities

that weren't affected by the droughts are masked out. **a** 2005 rainfall, **b** 2005 deforestation, **c** 2005 fire, **d** 2005 aerosol, **e** 2010 rainfall, **f** 2010 deforestation, **g** 2010 fire, **h** 2010 aerosol. Red shades show positive z values of local coefficients, while blue shades show negative z values of local coefficients - the darker the shade the stronger the relationship. Z values of local coefficients were generated in GWR4 and mapped using ArcGIS 10.

a) SOCIO model 2005 - Rainfall

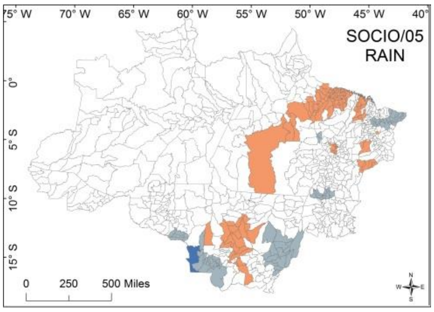

b) SOCIO model 2005 - Deforestation

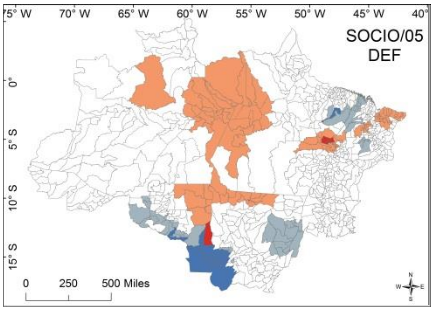

c) SOCIO model 2005 - Fire

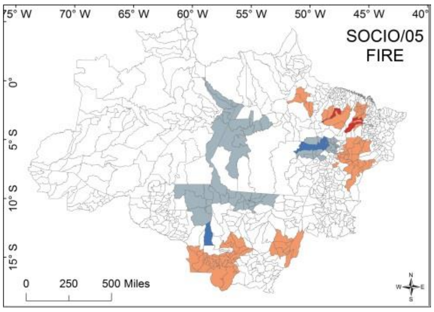

d) SOCIO model 2005 - Aerosol

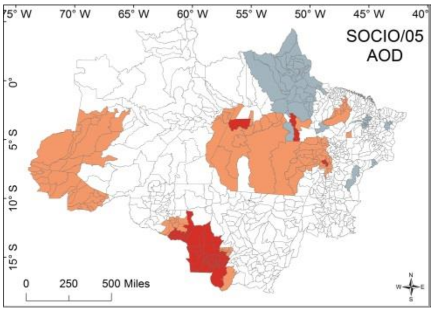

e) SOCIO model 2005 - Population

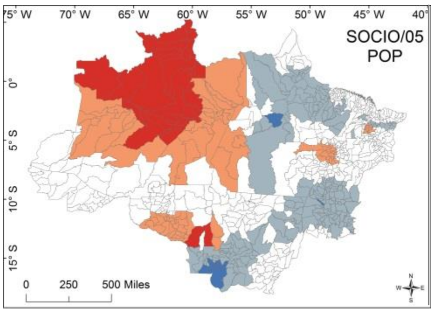

g) SOCIO model 2010 - Rainfall

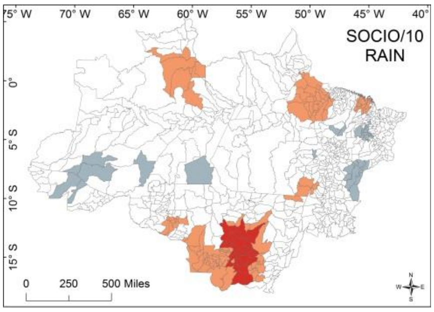

h) SOCIO model 2010 - Deforestation

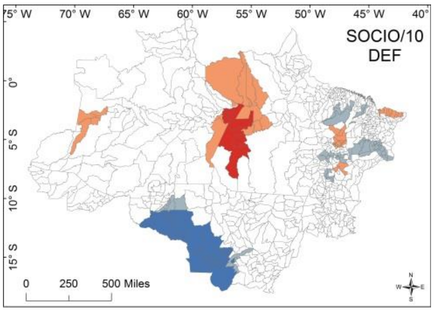

i) SOCIO model 2010 - Fire

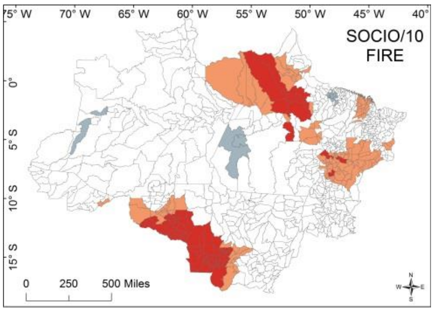

j) SOCIO model 2010 - Aerosol

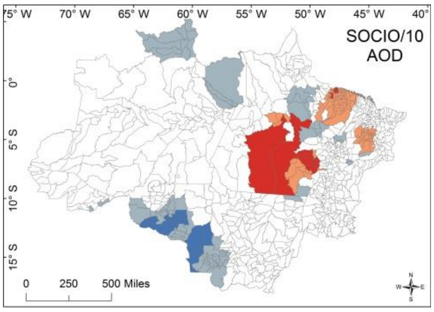

k) SOCIO model 2010 - Population

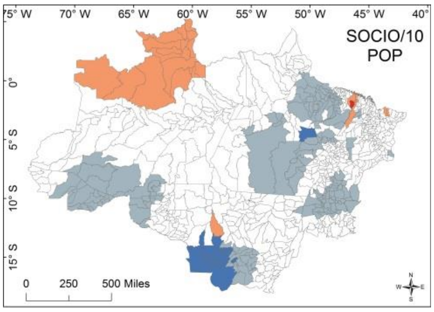

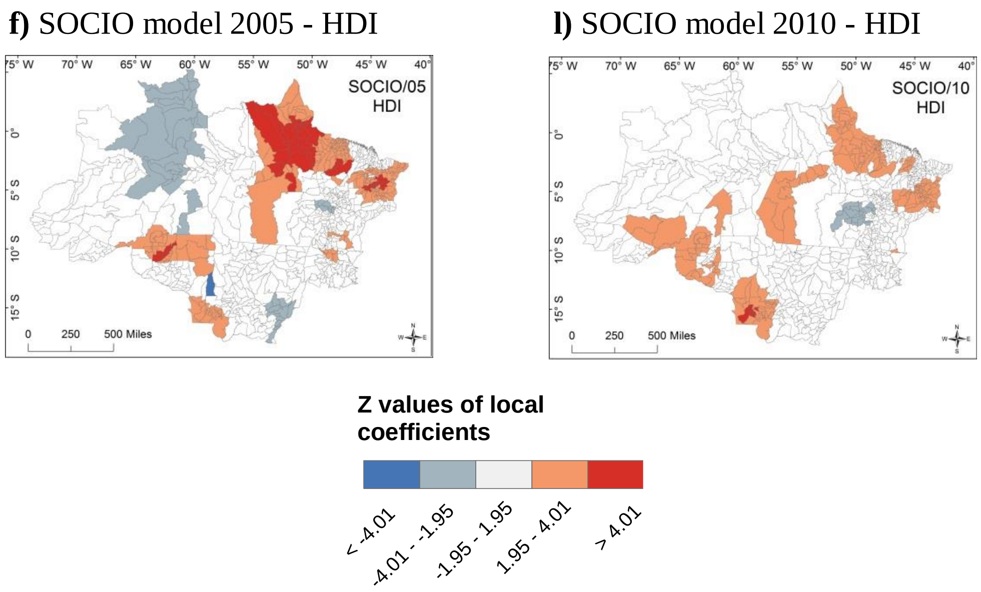

Figure S10| Significant values with a confidence level greater than 95% for the SOCIO model for the whole Brazilian Legal Amazon. Non-significant municipalities and municipalities that were not affected by the droughts are masked out. **a** 2005 rainfall, **b** 2005 deforestation, **c** 2005 fire, **d** 2005 aerosol, **e** 2005 population density, **f** 2005 HDI, **g** 2010 rainfall, **h** 2010 deforestation, **i** 2010 fire, **j** 2010 aerosol, **k** 2010 population density, **l** 2010 HDI . Red shades show positive z values of local coefficients, while blue shades show negative z values of local coefficients - the darker the shade the stronger the relationship. Z values of local coefficients were generated in GWR4 and mapped using ArcGIS 10.

a) SOCIO model 2005 - Rainfall

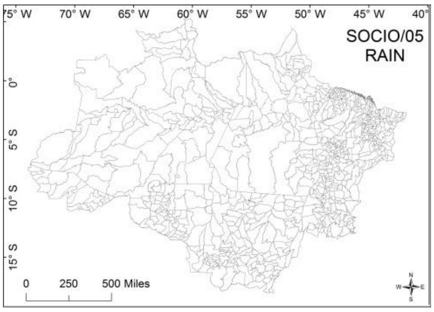

b) SOCIO model 2005 - Deforestation

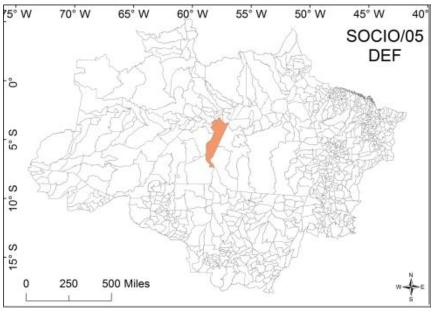

c) SOCIO model 2005 - Fire

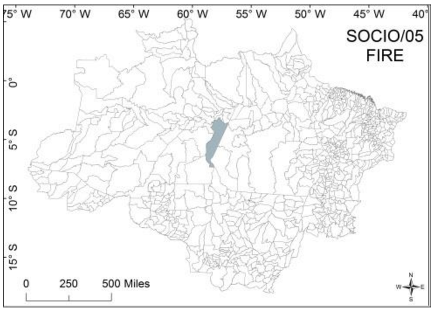

d) SOCIO model 2005 - Aerosol

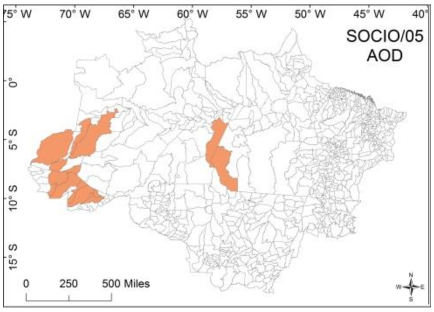

e) SOCIO model 2005 - Population

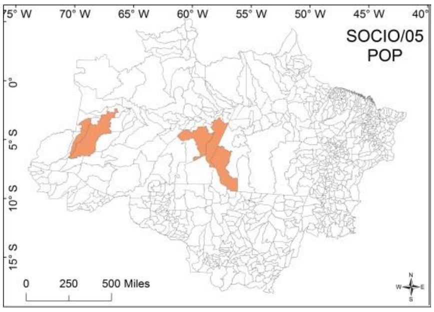

g) SOCIO model 2010 - Rainfall

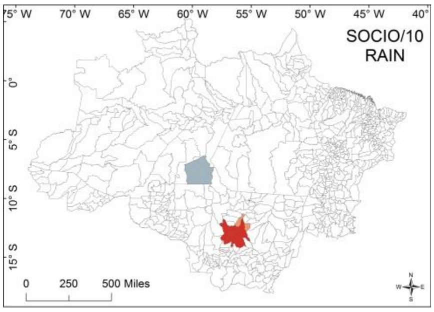

h) SOCIO model 2010 - Deforestation

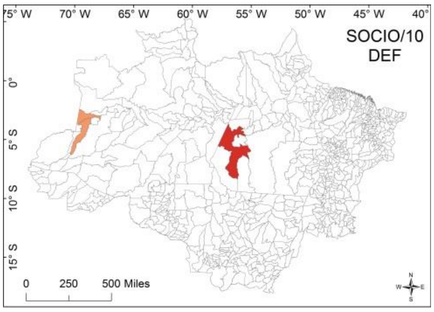

i) SOCIO model 2010 - Fire

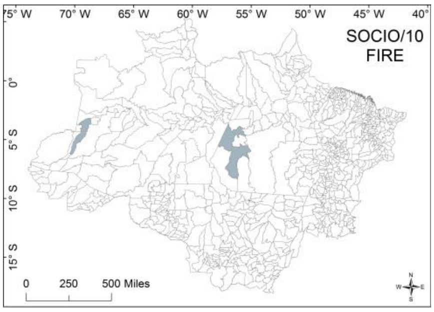

j) SOCIO model 2010 - Aerosol

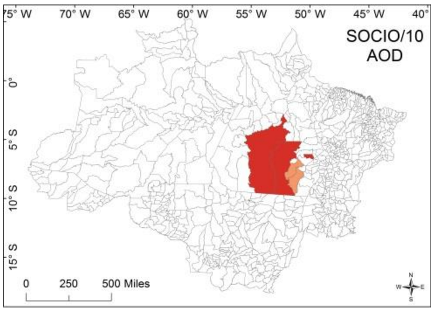

k) SOCIO model 2010 - Population

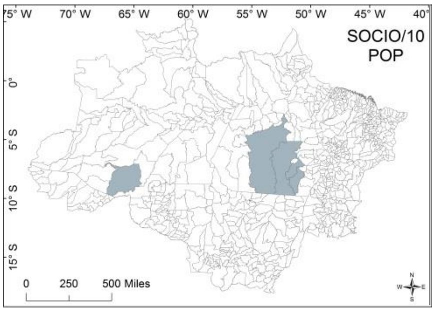

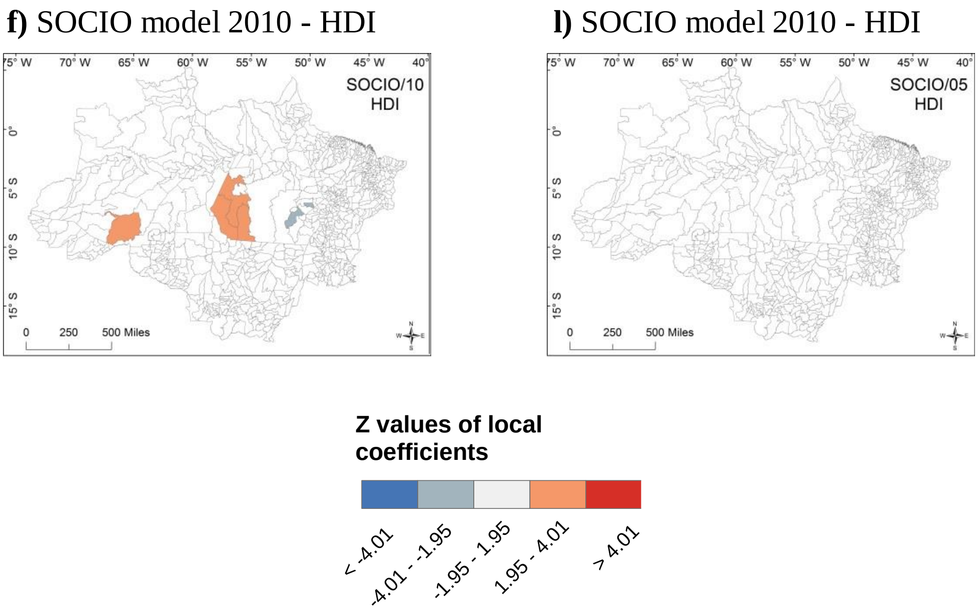

Figure S11| Significant values with a confidence level greater than 95% for the SOCIO model in severely affected (100mm rainfall & ≥ 2sd) drought municipalities. Non-significant municipalities and municipalities that were not severely affected by the droughts are masked out. **a** 2005 rainfall, **b** 2005 deforestation, **c** 2005 fire, **d** 2005 aerosol, **e** 2005 population density, **f** 2005 HDI, **g** 2010 rainfall, **h** 2010 deforestation, **i** 2010 fire, **j** 2010 aerosol, **k** 2010 population density, **l** 2010 HDI . Red shades show positive z values of local coefficients, while blue shades show negative z values of local coefficients - the darker the shade the stronger the relationship. Z values of local coefficients were generated in GWR4 and mapped using ArcGIS 10.

Supplementary References

1 Shuttleworth, W. J. *et al.* Micrometeorology of Temperate and Tropical Forest [and Discussion]. *Phil. Trans. R. Soc. B Biol Sci.* **324**, 299-334 (1989).

2 da Rocha, H. R. *et al.* Seasonality of water and heat fluxes over a tropical forest in eastern Amazonia. *Ecol Appl.* **14**, 22-32 (2004).

3 Wood, C. H., Skole, D. in *People and Pixels: Linking Remote Sensing and Social Science* (ed D. Liverman, Moran, E.F., Rindfuss, R.R., Stern, P.C.) Ch. 4, 70 - 93 (The National Academies Press, 1998).

4 Fotheringham AS, B. C., Charlton ME *Geographically Weighted Regression: The Analysis of Spatially Varying Relationships*. (Wiley, 2002).

5 Lovett, A. & Flowerdew, R. Analysis of count data using poisson regression*. *Prof Geogr.* **41**, 190-198 (1989).

6 Nakaya, T., Fotheringham, A. S., Brunsdon, C. & Charlton, M. Geographically weighted Poisson regression for disease association mapping. *Stat. Med.* **24**, 2695-2717 (2005).

7 DATASUS. (Ministério da saude / DATASUS - Departamento de Informática do SUS, 2011).

8 NASA. 2010. *Monthly 0.25^o x 0.25^o TRMM and other sources rainfall (TRMM product 3B43v6)* [Online]. NASA Distrib. Active Arch. Cent., Goddard Space Flight Cent. Earth Sci., Greenbelt, Md. Available: http://disc.gsfc.nasa.gov/data/datapool/TRMM_DP/01_Data_Products/02_Gridded/07_Monthly_Other_Data_Source_3B_43/ [Accessed 18/10/2010].

9 INPE. 2012. *INPE PRODES* [Online]. http://www.dpi.inpe.br/prodesdigital/prodes.php: Ministério da Ciência e Tecnologia. [Accessed 01/11/2012].

10 INPE. 2012. *INPE Queimadas* [Online]. http://www.dpi.inpe.br/proarco/bdqueimadas/ Ministério da Ciência, Tecnologia e Inovação e Ministério do Meio Ambiente. [Accessed 01/11 2012].

11 NASA. 2011. *Atomsphere Aerosol optical depth at 0.55µm MOD08 M3.005* [Online]. http://ladsweb.nascom.nasa.gov/data/search.html. [Accessed 03/10/2011].

12 Instituto Brasileiro de Geografia e Estatística. 2010. Indicadores [Online] *http://www.ibge.gov.br/home/mapa_site/mapa_site.php#populacao* [Accessed 01/07/2011].

13 Mallol, J. *et al.* Cambios en la prevalencia de asma en escolares chilenos entre 1994 y 2002: International Study of Asthma and Allergies in Childhood (ISAAC) - Chile phases I and III. *Rev. Med. Chil.* **135**, 580-586 (2007).

14 Sistema Firjan. 2013. *Sistema Firjan Índice FIRJAN de Desenvolvimento Municipal* [Online]. http://www.firjan.org.br/ifdm/ Sistema Firjan. [Accessed 01/03/2012].

15 Mallol, J. *et al.* Prevalence of asthma symptoms in Latin America: The international study of asthma and allergies in childhood (ISAAC). *Pediatr. Pulmonol.* **30**, 439-444 (2000).

16 Gonçalves-Silva, R. M. V., Valente, J. G., Lemos-Santos, M. G. F. & Sichieri, R. Tabagismo no domicílio e doença respiratória em crianças menores de cinco anos. *Cad. Saude Publica.* **22**, 579-586 (2006).
